# Characterization of Tiered Psychological Distress Phenotypes in an Orthopaedic Sports Population

**DOI:** 10.3390/ijerph22060914

**Published:** 2025-06-09

**Authors:** Billy I. Kim, Nicholas J. Morriss, Taylor P. Stauffer, Julia E. Ralph, Caroline N. Park, Trevor A. Lentz, Brian C. Lau

**Affiliations:** Duke Department of Orthopaedic Surgery, Duke University Medical Center, 200 Trent Drive, Durham, NC 27710, USA; billy.kim@duke.edu (B.I.K.); nicholas.morriss@duke.edu (N.J.M.); taylor.stauffer@duke.edu (T.P.S.); caroline.park@duke.edu (C.N.P.); trevor.lentz@duke.edu (T.A.L.); brian.lau@duke.edu (B.C.L.)

**Keywords:** psychological distress, phenotypes, musculoskeletal pain, OSPRO

## Abstract

Psychological distress and musculoskeletal pain are interconnected with poor functional outcomes. This study sought to classify common phenotypes of psychological distress in an orthopaedic sports population and assess differences in functional outcomes using the Prediction of Referral and Outcome (OSPRO-YF) tool. This was a cross-sectional study on 411 operative patients from a single sports surgeon’s clinical practice with completed OSPRO-YF questionnaires. Latent class analysis was employed to construct distress phenotypes based on binary measures for 11 single-construct psychological questionnaires, spanning two negative and one positive domains of pain-associated psychological distress. Functional outcome measures, including numerical pain scores, the Patient-Reported Outcomes Measurement Information System (PROMIS), the Single Assessment Numeric Evaluation (SANE) American Shoulder and Elbow Surgeons Score (ASES), and the International Knee Documentation Committee Subjective Knee Form (IKDC), were compared. Four psychological distress clusters were derived: low distress (LD-1; *n* = 111), low self-efficacy (LS-2; *n* = 101), negative pain coping, low self-efficacy (NP-3; *n* = 99), and high distress (HD-4; *n* = 100), with increasing yellow flags proceeding from LD-1 to HD-4. The mean numerical pain scores were highest in HD-4 and lowest in LD-1 and LS-2 (4.6 vs. 2.7 and 2.0, respectively; *p* < 0.001). The PROMIS depression scores were highest in HD-4 compared to NP-3, LS-2, and LD-1 (57.0 vs. 48.9 vs. 45.6 vs. 46.0; *p* < 0.001). Phenotyping patients based on OSPRO-YF distress indicators provides an initial framework of the psychological distress burdening the average orthopaedic sports surgical patient population and may aid in targeted psychological treatments.

## 1. Introduction

Despite advances in surgical techniques and evidence-based rehabilitation protocols, many patients report chronic pain and lasting dysfunction after orthopaedic sports surgeries [1,2]. There is a complex interplay between psychological distress and musculoskeletal pain [3,4,5,6]. A growing body of literature has demonstrated that psychosocial factors such as emotional distress, poor pain coping mechanisms, and reduced resiliency are associated with increased pain intensity, physical disability, poor functional outcomes, and prolonged postoperative opioid use in patients with orthopaedic sport-related injuries [7,8,9,10,11,12]. Growing awareness of the importance of psychological distress after surgery, coupled with the availability of psychological questionnaires that assess multiple domains of psychological distress (i.e., depression, fear avoidance, and self-efficacy), have created opportunities to better understand and manage these characteristics post-surgery. One such opportunity is through psychological phenotyping. Phenotyping facilitates the identification of patient subgroups that would most benefit from tailored, psychologically informed rehabilitation interventions to improve post-surgical outcomes.

However, there is a paucity of literature describing the psychological profiles that exist in an orthopaedic sports population, which is typically younger and healthier than the average osteoarthritis population [13]. One cross-sectional study deployed abbreviated questionnaires and sought to identify psychological distress phenotypes in 137 patients with shoulder pathologies, albeit limited by the sample size and the breadth of the psychological domains covered [14]. Deepening our understanding of the psychological phenotypes that exist in the orthopaedic sports population is important in identifying patients at higher risk for suboptimal outcomes and providing targeted multimodal therapy to improve patient care [15,16]. Additionally, it is important to identify these phenotypes in a surgical population in order to optimize both preoperative expectations and postoperative recovery. Therefore, the aim of this study was to identify unique psychological distress phenotypes in a sports medicine clinic at a large institution, leveraging the use of the Optimal Screening or Prediction of Referral and Outcome (OSPRO-YF), a multidimensional psychological assessment tool that measures depression, anxiety, self-efficacy, pain catastrophizing, and fear avoidance [17]. The authors hypothesized that a sports medicine population would result in fewer patients classified into high psychological distress phenotypes in comparison to older, hip and knee osteoarthritis psychological phenotypes, as evaluated in prior studies [17].

## 2. Methods

### 2.1. Cohort Selection

Institutional review board approval was obtained prior to conducting this retrospective study. Utilizing the institution’s electronic health record (EHR) database, we identified 433 patients who had completed a 10-item OSPRO-YF survey preoperatively or at the first postoperative visit between March 2020 and June 2022 and underwent an orthopaedic procedure by a single, fellowship-trained surgeon at a large academic institution. New patients were invited to complete the 10-item OSPRO-YF tool, a concise assessment tool that detects yellow flags for 11 single-construct psychological questionnaires that span 3 separate domains of pain-associated psychological distress (2 related to vulnerability and 1 to resilience) [17]. Yellow flagging is performed using OSPRO-YF tool score estimates, and patients receive flags if their score estimate is above the 75th percentile for negative psychological domains or below the 25th percentile for positive domains. The 11 psychological constructs (validated questionnaire), for which scores are estimated, include depression (Patient Health Questionnaire-9; PHQ-9), anxiety (State-Trait Anxiety Inventory; STAI), anger (State-Trait Anger Expression Inventory; STAXI), fear avoidance beliefs for physical activities and for work (Fear Avoidance Beliefs Questionnaire; FABQ-PA and FABQ-W, respectively), pain catastrophizing (Pain Catastrophizing Scale; PCS), kinesiophobia (Tampa Scale of Kinesiophobia-11; TSK-11), pain-related anxiety (Pain Anxiety Symptoms Scale-20; PASS-20), pain self-efficacy (Pain Self-Efficacy Questionnaire; PSEQ), rehabilitation self-efficacy (Self-Efficacy for Rehabilitation Outcome Scale; SER), and pain acceptance (Chronic Pain Acceptance Questionnaire; CPAQ). Patient survey responses were entered online in the medical chart prior to the visit on a personal device or using a tablet device during the visit, and surveys were completed by patients on a voluntary basis. Although the OSPRO-YF tool is offered to all patients at this surgeon’s clinic, the decision was made to include only operative patients in this analysis such that the derived psychological profiles were reflective of patients with significant orthopaedic pathologies meeting the indications for surgical treatment. In this manner, the psychologically informed treatment recommendations derived from the characteristics of our psychological distress phenotypes may best be applied to other cohorts of surgical patients. In this cohort, patients underwent mainly sports-related orthopaedic procedures such as shoulder labrum or rotator cuff repair, capsular repair or reconstruction, knee ligamentous or meniscus repair/reconstruction, chondroplasty, open reduction internal fixation, and tenodesis or tenotomy. Nine patients with incomplete OSPRO-YF surveys were also excluded. Ultimately, 411 patients with completed OSPRO-YF surveys were included for subsequent analysis.

### 2.2. Patient and Survey Data

Data on patient demographics and baseline characteristics, including age, race, ethnicity, sex, body mass index (BMI), current smoking status, and anesthesia type (general vs. other), were collected from the institutional EHR. Preoperative patient-reported outcomes such as numerical pain rating, Single Assessment Numeric Evaluation (SANE), Patient-Reported Outcomes Measurement Information System (PROMIS), American Shoulder and Elbow Surgeons Score (ASES), and International Knee Documentation Committee (IKDC) were also obtained. Preoperative survey data on pain, SANE, PROMIS, ASES, and IKDC were available for approximately half of the patients in this cohort (range: 49.3–57.8%).

### 2.3. Latent Class Analysis

A latent class analysis was performed on the 11 OSPRO yellow flag binary measures (yes or no) using the poLCA (version1.6.0.1, R package, R Core Team, Vienna, Austria) to derive classes or “phenotypes” of psychological distress in this patient cohort [18]. This probability-based classification method assigned patients to classes based on their highest posterior probability. The number of classes (*k*) was determined in a similar fashion to Lentz et al. [17], deriving psychological distress phenotypes in hip and knee osteoarthritis patients [19]. In brief, we considered model fit measurements such as the Akaike and Bayesian information criteria (AIC and BIC), likelihood ratio chi-squared statistic (L^2^), and model entropy (Table 1). Entropy was used to calculate the classification accuracy and quality, with values closer to 1 indicating clearer classification. The equation for model entropy utilized can be seen below:

$$Entropy=1-\frac{\sum_{i-1}^{N}\sum_{k-1}^{K}{\hat{p}}_{ik}\log{(\hat{p}}_{ik})}{N\log(K)}$$

We also ensured adequate class representation (each group containing > 10% of the total cohort) and the clinical interpretability of the reported probabilities of reporting yellow flags on various questionnaires. The selected model prioritized the AIC and BIC values, significant L^2^ test, and high entropy. A four-class system showed the most favorable connections of these metrics (Table 2) [20].

### 2.4. Statistical Analysis

Continuous variables were represented as means and standard deviations, and categorical variables were represented as counts and proportions. The normality of continuous variables was assessed visually using histogram plots and quantitatively with the Shapiro–Wilk normality test. Univariable analysis was performed using analysis of variance (ANOVA) or chi-squared tests as appropriate. Patient-reported outcomes were found to be normally distributed and were subsequently analyzed with ANOVA, with post hoc comparisons performed via Tukey’s honestly significant difference (TSD) and pairwise chi-squared tests. The post hoc testing *p*-values were subsequently adjusted for multiple comparisons using the Benjamini–Hochberg (BH) method.

### 2.5. Transparency and Openness

Patient sample selection, exclusion criteria, data manipulations, and measures are reported in the above sections. All univariable statistical analyses were performed in R version 3.6.1 (R Foundation, Vienna, Austria) using the *stats* package version 3.6.2. *p*-value < 0.05 was considered to indicate a statistically significant difference. This study’s design and its analysis were approved by an IRB.

## 3. Results

**Figure 1:**
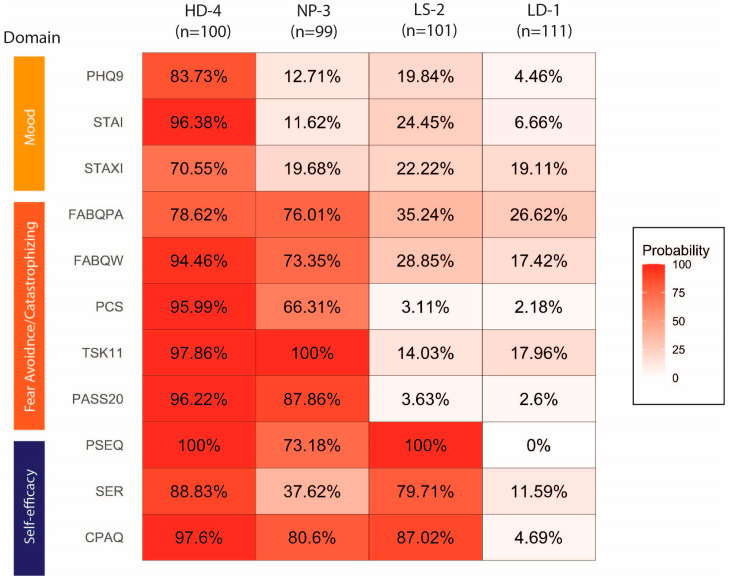
Tiered phenotype classification system.

The latent cluster analysis produced four classes based on the model fit characteristics, which considered the AIC and BIC values, significant L^2^, and model entropy, in addition to classes possessing > 10% of the population in each. These classes were labeled as the following, based on the estimated probabilities of obtaining yellow flags on individual psychological questionnaires: Class 1—low distress (LD-1); Class 2—low self-efficacy (LS-2); Class 3—negative pain coping, low self-efficacy (NP-3); and Class 4—high distress (HD-4). This classification method produced a tiered system of phenotypes, in which each increasing class raised yellow flags in an additional domain of psychological distress (Figure 1). In brief, HD-4 had yellow flags in both negative domains (mood and fear avoidance/catastrophizing) and the positive domain (resiliency). NP-3 differed from HD-4 by the absence of yellow flags in the mood domain. In comparison, LS-2 did not have yellow flags in any negative domain, but did have yellow flags in the positive, resiliency domain. Finally, LD-1 was our low-distress class, with relatively low probabilities of raising yellow flags in any domain. There were comparably similar numbers of patients in each class (n = 100, 99, 101, 111 for classes HD-4, NP-3, LS-2, LD-1, respectively).

In our sports patient population, LS-2 had the lowest mean patient age (39.5 yrs, sd: 18.2), while LD-1 (low distress) had the highest mean age (45.5, sd: 17.9; *p* = 0.028) (Table 3). The racial distribution across classes demonstrated a stepwise decrease in the proportion of White/Caucasian patients and, reversely, a stepwise increase in the proportion of Black/African American with increasing psychological distress classes (*p* < 0.001). A small group of “Other/Mixed/Not reported” patients also demonstrated a trend towards higher prevalence in higher-distress classes. There was no significant difference in ethnic distribution across classes. Smoking prevalence was also associated with increasing class numbers, with 14.5% of HD-4 patients compared to 2.7% of LD-1 patients being current smokers (*p* = 0.032). There were no statistically significant differences in sex, anesthesia type (general vs. other), or procedure type between classes.

Evaluating the patient-reported measures, we found that HD-4 had the highest mean numerical pain rating scores (4.7, sd 3.3). Compared to HD-4, LD-1 and LS-2 patients had lower mean pain scores in the pairwise post hoc analysis (2.6, sd 2.5, *p* < 0.001 and 2.0, sd 2.6, *p* < 0.001, respectively). Regarding patients undergoing surgery for shoulder pathologies (i.e., rotator cuff or labral repair), HD-4 and NP-3 patients had lower mean ASES scores (35.5 and 40.2, respectively) than LS-2 and LD-1 (60.4 and 52.3; *p* = 0.029), although the pairwise comparisons did not identify statistically significant differences. No significant differences were found in the ASES scores for the unaffected shoulder or for the IKDC scores. The PROMIS category scores revealed similar class trends, with HD-4 and NP-3 having higher mean pain interference scores (*p* = 0.007) and higher mean sleep disturbance scores (*p* < 0.001). The mean PROMIS depression scores were highest in HD-4 compared to NP-3, LS-2, and LD-1 (57.1 vs. 48.9 vs. 45.6 vs. 46.0; *p* < 0.001). Statistically significant differences from the post hoc pairwise comparisons can be found in Table 4.

## 4. Discussion

The present study identified four tiered psychological distress phenotypes in patients presenting for sports-related orthopaedic shoulder, knee, and foot and ankle pathologies. Compared to previous patient profiling studies in patients suffering from knee and hip osteoarthritis, there are fewer patients falling into the respective high-distress classes. This present study offers a conceptual framework to triage surgical patients according to their psychologic risk factors, with the goal of optimizing their psychological recovery after surgery.

Concise, multidimensional psychological assessment tools allow physicians to better characterize the heterogeneity of patient profiles in an orthopaedic setting [21,22,23]. The psychological distress phenotypes generated in our analysis are best interpreted in the context of phenotypes from similar patient profiling studies [14,19]. Broadly speaking, our phenotypes were similar in size (each comprising approximately a quarter of the patients, 24.1-27.0%) and demonstrated a unique, tiered pattern in which one additional psychological domain was “yellow-flagged” with rising class numbers. This was in contrast with the phenotypes identified in 1,239 patients with hip and knee osteoarthritis (OA), where a specific “negative pain coping” phenotype without yellow flags in the positive pain coping (self-efficacy) domain exists. Furthermore, over half of the OA cohort (*n* = 646, 52%) fell into the high-distress group, in comparison to only a quarter of our patients falling into the HD-4 group, likely related to the significant psychological burden of chronic pain, which was less prevalent in our younger sports population [24,25,26]. Comparatively, Miner et al. identified only 5 of 137 total patients with upper-extremity illness falling into their high-distress equivalent, “Phenotype 4”, with the majority (n = 77/137, 56%) of patients in their low-distress equivalent, “Phenotype 1” [14]. In our cohort, which included patients with shoulder pathologies (rotator cuff, labral, and capsular injury), we did not detect a disproportionate number of shoulder procedures in our low-distress class (Table 3, *p* = 0.615). The differences in our distress group proportions may be due to differences in the sample size, our study’s inclusion of only patients undergoing surgery, or survey method differences, with our study’s utilization of a multidimensional assessment tool that provided estimates across more psychological constructs.

Across our phenotypes, the patient-reported outcome measures depict a general trend toward worse functional scores in higher-distress classes, although the HD-4 and NP-3 classes demonstrated similar pain, function, and demographic profiles. The findings suggest that our analysis had sufficient resolution to identify a low-self-efficacy group that may be indifferentiable from the low-distress class when only assessing function- or pain-related measures but harbor a distinct psychological profile with a significant likelihood of raising yellow flags across self-efficacy and chronic pain acceptance measures. As our OSPRO-YF measurements were obtained either preoperatively or shortly after surgery at the patients’ first postoperative visit, we were unable to assess the long-term differences between these groups. For example, it may be that patients within the low-self-efficacy group are less likely to participate meaningfully in physical therapy during their postoperative rehabilitation, in turn leading to slower or overall worse functional recovery. Similarly, these patients may be more sensitive to ongoing postoperative pain and therefore less satisfied with their overall recovery. Further research on the temporal dynamicity of psychological profiles is warranted to better inform psychological interventions in recovery from musculoskeletal injury.

The utility of preoperative phenotyping is that, by classifying patients into four phenotype profiles, surgeons can offer specific, personalized treatments to address these psychological vulnerabilities, as outlined below for each phenotype.

**Figure 2:**
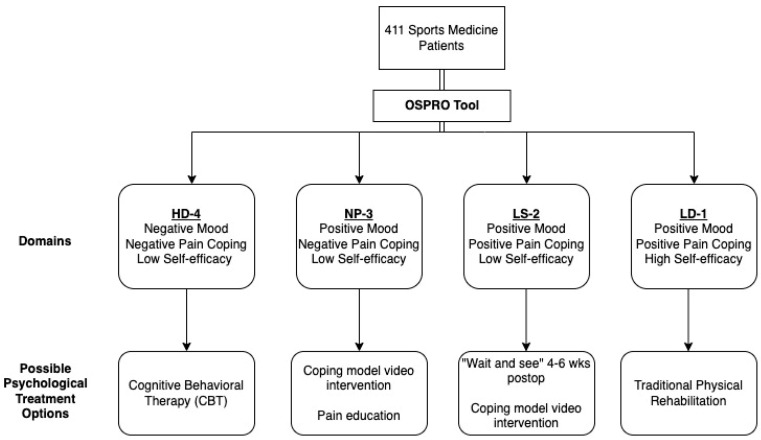
Phenotype outlines and suggested treatments.

(1) Patients in HD-4 may be most likely to benefit from early psychosocial interventions and more frequent monitoring. Cognitive behavioral therapy (CBT) is highly effective in reducing psychological distress following surgery and improves long-term scores for quality of life and reduced anxiety [27], but it is time-intensive and can be expensive for the patient. Because psychological interventions can be resource-intensive, it is important to limit referrals for CBT to patients that are most likely to benefit from it, such as patients with a HD-4 phenotype.
(2) Patients in NP-3 (poor pain coping and low self-efficacy) may benefit from lower-intensity interventions to enhance coping and self-efficacy, such as coping model video interventions, where models perform rehabilitation exercises, reflect on problems faced, and discuss strategies to overcome them in one preoperative and one postoperative video (total duration of 16 min) [28]. Coping model interventions are aimed at reducing anxiety and perceptions of pain, which is thought to improve self-efficacy and function in the postoperative rehabilitation period [29,30,31]. Studies have also demonstrated the efficacy of combining education led by a physical therapist with impairment-based interventions in lowering both pain catastrophizing scores and coping skill strategies [32,33,34,35]. Specifically, one-on-one pain neuroscience education (helping patients to re-conceptualize their pain from a neurobiological perspective) with information booklets is another option for lower-intensity intervention.
(3) Patients in LS-2 may be most likely to naturally transition to a low-distress phenotype over the course of routine rehabilitation as their confidence in physical activity improves. For this reason, these patients may benefit most from a “wait and see” approach, where traditional rehabilitation is offered and psychological distress is monitored over the first 4–6 weeks of rehabilitation to determine whether psychologically focused interventions are warranted.
(4) Finally, those in LD-1 would be the best candidates for traditional impairment-based rehabilitation, may not require any psychological monitoring, and may be good candidates for low-touch treatment pathways like self-directed care and telehealth [36] (Figure 2).

This conceptual framework for psychological support and treatment offered to patients falling into different classes is visualized in Figure 2.

There are several limitations to this study. Notably, the 10-item OSPRO-YF, developed through iterative item reduction from a 136-item pool, produces accurate estimates for individual questionnaires but is not equivalent to completing full individual psychological questionnaires, which limits our profiling accuracy. Additionally, yellow flags are population-based labels (below 25th or above 75th percentiles) and may not always reflect clinically significant thresholds. The phenotypes discerned from our study, with questionnaires collected pre- or postoperatively at patients’ first visits, should only be interpreted as static profiles, as the temporal dynamicity of the OSPRO-YF responses has not been evaluated. Therefore, the timing of survey administration is a potential cofounder in this study. As our study lacks a follow-up, we are unable to correlate the phenotypes with the recovery trajectories, which could be considered in a future investigation. Lastly, our single-surgeon, single-institution cohort was selected to minimize heterogeneity in the data collection methods, which inherently affects the generalizability of this study’s findings. Our cohort consisted of 411 patients, which, while large, may not fully capture the diversity of this population. Nevertheless, with the ability of our clustering analysis to detect distinct psychological profiles, we believe that our study findings provide novel contributions to the current understanding of psychological pain distress phenotypes in the orthopaedic sports population.

## 5. Conclusions

This study describes the novel characterization of tiered psychological distress phenotypes in an orthopaedic sports population. Similarly to prior studies, we detected high- and low-distress groups, although we found relatively fewer patients in the high-distress groups, as seen in hip and knee osteoarthritis cohorts. The present study also found racial disparities across psychological phenotypes, emphasizing the importance of addressing the increased psychological burden in racial minority groups, even in a relatively young and healthy orthopaedic sports population. This study provides a framework for the characterization of the heterogeneity of patient psychological profiles and allows sports medicine practitioners to better understand this population. Providers should consider implementing new classification systems, such as the one described in this study, to optimize psychological interventions offered to patients after surgery, particularly those facing higher levels of distress.

## Figures and Tables

**Table 1:** Model fit parameters for number of k classes.

Model—Number of Classes (*k*)	df	BIC	AIC	Likelihood Ratio	Entropy
**1**	400	6062.53	6073.53	2454.8055	-
**2**	388	4887.78	4910.78	1207.8278	0.931
**3**	376	4646.53	4681.53	894.3603	0.956
4 *	**364**	**4509.19**	**4556.19**	**684.7953**	**0.929**
**5**	352	4463.11	4522.11	566.4881	0.952
**6**	340	4444.32	4515.32	475.476	0.911

* Ideal classification system balances high entropy (close to 1) and a high likelihood ratio with lower BIC and AIC values.

**Table 2:** Model class membership for number of k classes.

Model—Number of Classes (k)	Class 1	Class 2	Class 3	Class 4	Class 5	Class 6
**2**	43.80%	56.20%	-	-	-	-
**3**	42.09%	28.22%	29.68%	-	-	-
4 *	**24.33%**	**24.09%**	**24.57%**	**27.01%**	**-**	**-**
**5**	18.20%	9.25%	22.87%	24.33%	25.30%	
**6**	25.30%	18.00%	8.52%	6.60%	18.25%	23.36%

k = number of classes. * Ideal model has all classes with relatively similar numbers of patients.

**Table 3:** Patient demographics, baseline characteristics, and procedures.

	HD-4	NP-3	LS-2	LD-1	*p*-Value
***N***	100	99	101	111	
**Age, mean (sd)**	39.84 (17.52)	44.42 (18.32)	39.49 (18.19)	45.49 (17.90)	**0.028**
**Race, *n* (%)**					**<0.001**
**Caucasian/White**	45 (45.0)	62 (62.6)	72 (71.3)	90 (81.1)	
**Black or African American**	43 (43.0)	27 (27.3)	18 (17.8)	16 (14.4)	
**Other/Mixed/Not Reported**	12 (12.0)	10 (10.1)	11 (10.9)	5 (4.5)	
**Ethnicity = Hispanic (%)**	31 (47.0)	29 (50.0)	39 (63.9)	40 (56.3)	0.235
**Male Sex, *n* (%)**	4 (4.2)	7 (7.4)	5 (5.2)	5 (4.8)	0.784
**BMI (kg/m^2^), mean (sd)**	31.30 (7.61)	29.60 (7.37)	28.43 (6.17)	28.94 (6.35)	0.084
**Current Smoker, *n* (%)**	10 (14.5)	7 (10.3)	3 (4.3)	2 (2.7)	**0.032**
**General Anesthesia (vs. Other), *n* (%)**	11 (16.4)	8 (11.8)	4 (5.9)	7 (9.3)	0.247
**Procedure, *n* (%)**	-	-	-	-	0.458
**-**	-	-	-	-	**Totals**
**AC Joint/Clavicle Resection**	12 (2.9)	1 (1.0)	4 (4.0)	2 (2.0)	5 (4.5)
**ACL, PCL, or Other Ligamentous Repair**	63 (15.3)	16 (16.0)	14 (14.1)	18 (17.8)	15 (13.5)
**LUA/MUA**	2 (0.5)	1 (1.0)	1 (1.0)	0 (0.0)	0 (0.0)
**Meniscus/Chondroplasty**	145 (35.3)	37 (37.0)	37 (37.4)	32 (31.7)	39 (35.1)
**MPFL Recon**	12 (2.9)	3 (3.0)	2 (2.0)	5 (5.0)	2 (1.8)
**ORIF**	43 (10.5)	12 (12.0)	9 (9.1)	11 (10.9)	11 (9.9)
**Other/Unspecified**	4 (1.0)	0 (0.0)	0 (0.0)	2 (2.0)	2 (1.8)
**Rotator Cuff Repair**	60 (14.6)	13 (13.0)	20 (20.2)	14 (13.9)	13 (11.7)
**Shoulder Labral/Capsule Repair**	33 (8.0)	13 (13.0)	5 (5.1)	7 (6.9)	8 (7.2)
**Tenodesis/Tenotomy**	37 (9.0)	4 (4.0)	7 (7.1)	10 (9.9)	16 (14.4)

Abbreviations: HD-4 = high distress class 4; NP-3 = negative pain coping, low self-efficacy class 3; LS-2 = low self-efficacy class 2; LD-1 = low distress class 1; BMI = body mass index; AC = acromioclavicular; ACL = anterior cruciate ligament; PCL = posterior cruciate ligament; I&D = irrigation and debridement; LOA/MUA = lysis of adhesions/manipulation under anesthesia; MPFL = medial patellofemoral ligament; ORIF = open reduction internal fixation.

**Table 4:** Preoperative patient-reported outcomes.

	HD-4	NP-3	LS-2	LD-1	*p*-Value	Post Hoc Pairwise Differences *
***N***	100	99	101	111		
**Pain, mean (sd)**	4.61 (3.30)	3.71 (2.95)	2.03 (2.62)	2.66 (2.50)	<0.001 *	4-2; 4-1; 3-2
**SANE, mean (sd)**	36.97 (26.88)	41.44 (26.14)	43.61 (31.17)	48.07 (26.19)	0.153	
**ASES, mean (sd)**	35.52 (21.49)	40.22 (26.03)	60.42 (28.31)	52.25 (15.57)	0.029 *	none
**ASES unaffected side, mean (sd)**	80.33 (27.17)	88.70 (20.77)	96.94 (4.76)	88.50 (21.61)	0.439	
**IKDC, mean (sd)**	30.76 (21.61)	33.43 (15.36)	37.06 (17.90)	38.25 (14.58)	0.334	
**IKDC unaffected side, mean (sd)**	77.80 (20.45)	79.40 (24.76)	84.33 (20.35)	88.67 (16.89)	0.188	
**PROMIS category, mean (sd)**						
** Physical Function**	33.58 (8.66)	35.02 (10.91)	36.35 (9.44)	38.19 (9.45)	0.091	
** Pain Interference**	67.08 (7.11)	63.49 (7.84)	60.84 (8.52)	61.46 (6.85)	<0.001 *	4-2; 4-1
** Sleep Disturbance**	59.36 (11.25)	53.90 (9.79)	51.59 (6.67)	52.18 (9.85)	0.007 *	none
** Depression**	57.05 (10.88)	48.91 (9.64)	45.61 (8.05)	45.96 (8.20)	<0.001 *	4-3; 4-2; 4-1

* statistically significant differences (*p* < 0.05) from pairwise comparisons after *p*-value correction for multiple comparisons. Abbreviations: HD-4 = high distress class 4; NP-3 = negative pain coping, low self-efficacy class 3; LS-2 = low self-efficacy class 2; LD-1 = low distress class 1; SANE = Single Assessment Numeric Evaluation; PROMIS = Patient-Reported Outcomes Measurement Information System; ASES = American Shoulder and Elbow Surgeons Score; IKDC = International Knee Documentation Committee.

## Data Availability

The original contributions presented in this study are included in the article. Further inquiries can be directed to the corresponding authors.

## Abbreviations

The following abbreviations are used in this manuscript:

OSPRO-YF	Optimal Screening or Prediction of Referral and Outcome—Yellow Flag
PROMIS	Patient-Reported Outcomes Measurement Information System
ASES	American Shoulder and Elbow Surgeons Score
IKDC	International Knee Documentation Committee Subjective Knee Form
EHR	Electronic Health Record
PHQ-9	Patient Health Questionnaire-9
STAI	State-Trait Anxiety Inventory
STAXI	State-Trait Anger Expression Inventory
FABQ	Fear Avoidance Beliefs Questionnaire
TSK-11	Tampa Scale of Kinesaphobia-11
PCS	Pain Catastrophizing Scale
SER	Self-Efficacy for Rehabilitation Outcome Scale
CPAQ	Chronic Pain Acceptance Questionnaire
BMI	Body Mass Index
SANE	Single Assessment Numerical Evaluation
AIC	Akaike Information Criterion
BIC	Bayesian Information Criterion
ANOVA	Analysis of Variance
HSD	Honestly Significant Difference
BH	Benjamini–Hochberg
HD-4	High Distress Class 4
LD-1	Low Distress Class
LS-2	Low Self-Efficacy Class 2
NP-3	Negative Pain Coping Class 3
